# Mechanistic Study of L-Rhamnose Monohydrate Dehydration Using Terahertz Spectroscopy and Density Functional Theory

**DOI:** 10.3390/molecules30051189

**Published:** 2025-03-06

**Authors:** Bingxin Yan, Zeyu Hou, Yuhan Zhao, Bo Su, Cunlin Zhang, Kai Li

**Affiliations:** 1 Department of Physics, Capital Normal University, Beijing 100048, China; 2220602058@cnu.edu.cn (B.Y.); 2220602073@cnu.edu.cn (Z.H.); 2220602038@cnu.edu.cn (Y.Z.); zclcnu@163.com (C.Z.); 2 Beijing Key Laboratory for Terahertz Spectroscopy and Imaging, Beijing 100048, China; 3 Key Laboratory of Terahertz Optoelectronics, Ministry of Education, Beijing 100048, China; 4 Department of Chemistry, Capital Normal University, Beijing 100048, China; likai123452024@163.com

**Keywords:** L-rhamnose, THz-TDS, dehydration, DFT, molecular vibration

## Abstract

L-rhamnose has recently gained attention for its potential to enhance vaccine antigenicity. To optimize its use as a vaccine adjuvant, it is important to understand the dehydration behavior of L-rhamnose monohydrate, which plays a critical role in modifying its physicochemical properties. This study investigated the spectroscopic characteristics of L-rhamnose and its monohydrate using terahertz time-domain spectroscopy (THz-TDS), Raman spectroscopy, and powder X-ray diffraction (PXRD). The results indicate that THz-TDS can more effectively distinguish the spectral features of these two compounds and can be used to reflect the structural changes in L-rhamnose monohydrate before and after dehydration. THz spectral data show that dehydration of L-rhamnose occurs at 100 °C, and continuous heating at 100 °C can complete the dehydration process within 6 min. Density functional theory (DFT) calculations revealed that water molecule vibrations significantly affect the THz absorption peaks. These findings indicate that removing water during dehydration causes substantial changes in molecular structure and dynamics. Overall, this study highlights the value of combining THz-TDS with DFT calculations to investigate the structures of carbohydrates and their hydrates, providing an accurate method for understanding the dehydration process and molecular interactions in hydrated systems. This approach holds significant importance for the development of effective vaccine adjuvants.

## 1. Introduction

L-rhamnose, a pivotal 6-deoxy sugar, is ubiquitous in a diverse array of natural products, glycoproteins, and structural polysaccharides, predominantly existing in its L-form [1]. As a major sugar component of the O-antigen, L-rhamnose enhances the antigenicity of vaccines targeting tumor-associated carbohydrate antigens (TACAs) when it is incorporated [2]. Notably, Sarkar et al. demonstrated that the conjugation of L-rhamnose with carbohydrate antigens effectively bolstered the immune response in mice, underscoring its potential in immunotherapy [3]. Furthermore, the inclusion of L-rhamnose as a fundamental component of the Mycobacterium tuberculosis cell wall presents an intriguing avenue for the development of novel anti-Mycobacterium tuberculosis drugs in the fight against tuberculosis [4]. Consequently, L-rhamnose emerges as a compound of substantial research significance and vast application potential in the realm of drug discovery and development.

**Figure 1:**
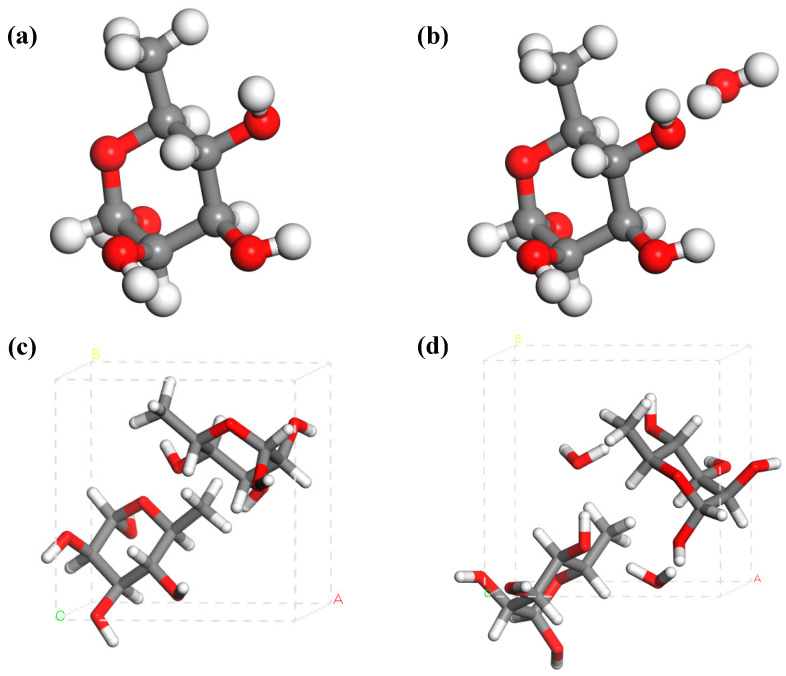
Molecular structure diagrams of L-rhamnose (**a**) and rhamnose monohydrate (**b**), and unit cell diagrams of L-rhamnose (**c**) and L-rhamnose monohydrate (**d**). The white, gray, and red spheres represent hydrogen (H) atoms, carbon (C) atoms, and oxygen (O) atoms, respectively. ABC represents the unit cell parameters.

The crystalline polymorphism of L-rhamnose primarily exists in two forms: α and β. The α-form contains a molecule of crystalline water, which can be lost under specific conditions, leading to its transformation into the β-form or α,β-form of L-rhamnose [5]. As shown in Figure 1, L-rhamnose and its monohydrate both belong to the P21(4) space group crystal structure but differ in terms of crystal symmetry and unit cell parameters. Due to the presence of a molecule of crystalline water, α-type L-rhamnose is also known as L-rhamnose monohydrate. Water molecules enter L-rhamnose through the crystallization process, forming specific intermolecular interactions [6]. These interactions affect the crystal structure of anhydrous L-rhamnose, which in turn influences the physicochemical properties of L-rhamnose-based products, such as pharmaceuticals and food, particularly in terms of stability and dissolution rate [7]. Consequently, a thorough characterization of L-rhamnose and its hydrated states is imperative for optimizing their preparation and storage conditions. Thermal analysis techniques commonly used to study pharmaceutical hydrates, such as differential scanning calorimetry (DSC) and thermogravimetric analysis (TGA), have certain limitations. On one hand, during DSC and TGA measurements, the sample is typically subjected to a flow of dry gas (commonly N_2_), which may lead to potential measurement errors [8,9]. On the other hand, while these methods provide comprehensive information on the thermal events of the sample, they cannot directly offer insights into molecular-level structure or dynamics [10]. In this study, a sample was measured under vacuum conditions in the terahertz time-domain system (THz-TDS) to avoid the potential influence of nitrogen. Additionally, we utilized density functional theory (DFT) and THz-TDS spectroscopy to analyze the dehydration process of L-rhamnose monohydrate.

THz refers to electromagnetic waves with frequencies ranging from 0.1 to 10 THz, located between the microwave and infrared regions of the electromagnetic spectrum [10,11,12,13]. Due to the photon energy of THz radiation being close to the excitation energy levels of biomolecular rotations and vibrations, THz spectroscopy can detect these molecular motions. This characteristic makes it highly applicable in fields such as chemical analysis, biological detection, and drug development [14,15]. For example, Komandin et al. used THz spectroscopy to study the dielectric response of α-lactose monohydrate at different temperatures, identifying the temperature range in which the hydrate exists [16]. Moreover, THz spectroscopy is highly sensitive to molecular structures, making it effective in distinguishing carbohydrates and their hydrates. Takahashi et al. utilized THz spectroscopy combined with DFT calculations to analyze the molecular structure and vibrational modes of glucose and its monohydrate, yielding characteristic spectra for both compounds [17]. Zhang et al. investigated the low-frequency spectra of gallic acid (GA) and its monohydrate using THz time-domain spectroscopy, monitoring the dehydration process of GA monohydrate in real time [18]. Additionally, THz spectroscopy has shown unique advantages in the analysis of mixtures. For instance, Yan et al. employed THz spectroscopy for the qualitative and quantitative analysis of glucose hydrates and glycosides, successfully identifying the components of commercial glucose powders [19]. Yang, Wei, Li, and others have provided valuable insights into the use of THz spectroscopy for monitoring the dehydration processes of hydrates [20,21,22,23]. Due to the sensitivity of THz waves to water content and molecular structure, THz-TDS spectroscopy provides an effective method for reflecting the dehydration process of hydrates.

In this study, we employed THz-TDS to measure the characteristic spectra of L-rhamnose and its monohydrate within the 0.3–2.75 THz range. To ensure the accuracy of these results, we complemented the THz measurements with Raman spectroscopy and powder X-ray diffraction (PXRD). Given the high sensitivity of THz spectroscopy to molecular structure, we further utilized the THz-TDS system to investigate the changes in L-rhamnose monohydrate at different heating times, with a particular focus on its dehydration temperature and the duration of the process. Additionally, DFT was used to perform quantum chemical calculations for both samples. These results revealed that the inclusion of water molecules significantly alters the molecular vibrational modes, consequently affecting the characteristic THz spectra of the substances.

## 2. Results and Discussion

### 2.1. Solid THz Absorption Spectra of L-Rhamnose and Its Monohydrate

**Figure 2:**
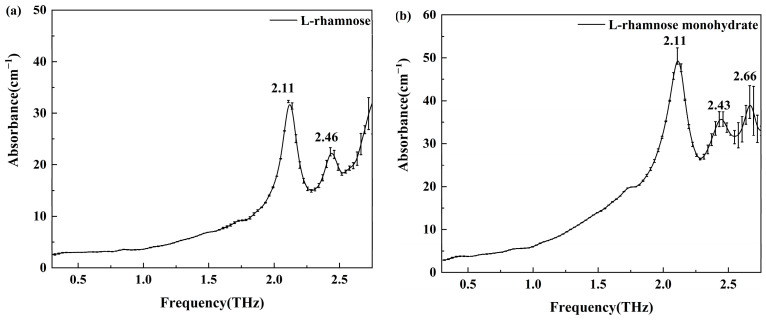
THz experimental absorption spectra and error bars of L-rhamnose (**a**) and L-rhamnose monohydrate (**b**) at 25 °C.

We utilized a THz-TDS system to assess solid tablet samples of L-rhamnose and its monohydrate, as shown in the THz absorption spectra in Figure 2. As illustrated in Figure 2a, L-rhamnose exhibits two distinct absorption peaks within the 0.3–2.75 THz range, with the most prominent peak at 2.11 THz and another at 2.46 THz. Figure 2b reveals three characteristic absorption peaks for L-rhamnose monohydrate: a strong peak at 2.11 THz, coinciding with that of L-rhamnose, and two weaker peaks at 2.43 and 2.66 THz. These findings underscore the utility of THz absorption spectroscopy in effectively distinguishing between L-rhamnose and its monohydrate based on their unique spectral characteristics.

### 2.2. Raman Spectra of L-Rhamnose and L-Rhamnose Monohydrate

To further explore whether there are specific molecular bonding differences between L-rhamnose and L-rhamnose monohydrate and to determine if these differences contribute to the variations observed in their THz absorption spectra, we conducted a Raman spectroscopy analysis.

**Figure 3:**
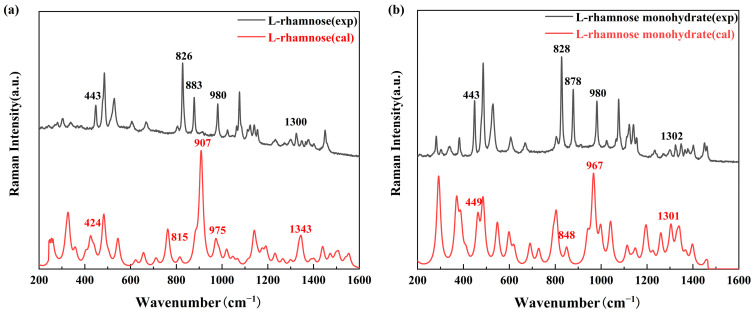
Comparison between experimental and calculated Raman spectra of L-rhamnose (**a**) and L-rhamnose monohydrate (**b**). Spectra are vertically offset for clarity.

Figure 3 shows a comparison of the experimental and calculated Raman spectra of L-rhamnose and L-rhamnose monohydrate in the 200–1600 cm^−1^ range. The findings reveal that both compounds generally exhibit similar scattering peak characteristics. More specifically, peaks in the 400–450 cm^−1^ and 810–900 cm^−1^ ranges correspond to the bending vibrations of the -OH group and the symmetric and asymmetric stretching vibrations, respectively. Additionally, peaks near 980 cm^−1^ and 1300 cm^−1^ are attributed to the torsional vibration of the -O- group and the wagging vibration of the -OH group, respectively. These regions are crucial for understanding the vibrational characteristics of both L-rhamnose and its monohydrate, highlighting their structural similarities in terms of functional group vibrations.

However, using Raman spectroscopy to clearly distinguish the intrinsic differences between L-rhamnose and L-rhamnose monohydrate remains a challenge, due to their close vibrational features.

### 2.3. PXRD of L-Rhamnose and L-Rhamnose Monohydrate

**Figure 4:**
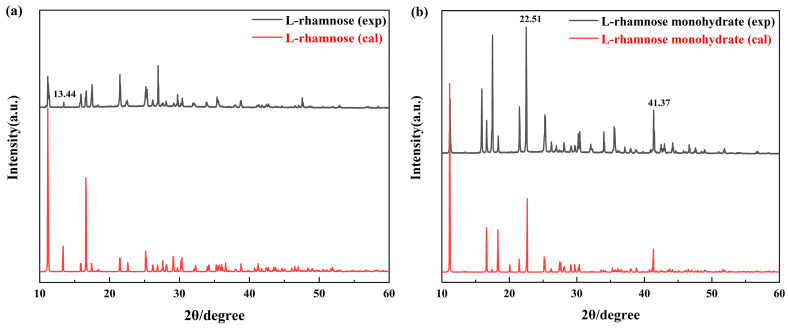
Comparison of PXRD experiment and calculated diffraction patterns of L-rhamnose (**a**) and L-rhamnose monohydrate (**b**) (with vertical spectral shift for clarity).

To assess whether the incorporation of water molecules into the L-rhamnose lattice leads to distinct unit cell structures, we performed a comparative analysis of the PXRD for L-rhamnose and its monohydrate. As illustrated in Figure 4a, L-rhamnose exhibits a diffraction peak at 13.44°, which is absent in L-rhamnose monohydrate. In contrast, the diffraction peaks of L-rhamnose monohydrate at 22.51° and 41.37° in Figure 4b are sharper and more pronounced compared to those of L-rhamnose. The appearance, disappearance, and enhancement of diffraction peaks indicate differences in the lattice structures of the two forms, suggesting that the introduction of water molecules significantly alters the crystal structure of L-rhamnose.

Additionally, the calculated PXRD in Figure 4a,b shows excellent agreement with the experimental spectra of L-rhamnose and its monohydrate. This strong consistency validates the structural model used in the computational simulations and confirms the rationality of the unit cell structure representing L-rhamnose and its monohydrate.

### 2.4. Evolution of THz Spectra During Dehydration Process

Given the importance of characterizing the dehydration process of L-rhamnose monohydrate and the high sensitivity of THz spectroscopy to structural changes, we employed THz spectroscopy to investigate the dehydration temperature and duration of the process in L-rhamnose monohydrate.

**Figure 5:**
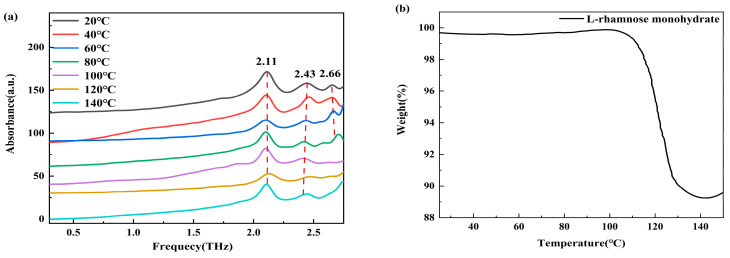
(**a**) THz spectra of L-rhamnose monohydrate at different temperatures (spectra are vertically offset for clarity); (**b**) TGA curve of L-rhamnose monohydrate.

Figure 5 illustrates the THz spectra of L-rhamnose monohydrate after maintaining the sample at various temperatures for 10 min. The spectra reveal changes clearly, including minor frequency shifts and the disappearance of specific absorption peaks during dehydration. The effects of the crystallization water on the THz spectral changes can be attributed to two primary factors. First, the reduction in the number of water molecules leads to a decrease in absorption intensity, but does not directly cause frequency shifts. Second, structural rearrangements during the dehydration process can result in peak shifts, peak disappearances, or the emergence of new peaks [24].

As shown in Figure 5a, the absorption peak at 2.66 THz gradually decreases from 20 °C to 100 °C and disappears, stabilizing between 100 °C and 140 °C. In conjunction with the TGA curve of L-rhamnose monohydrate in Figure 5b, a significant weight loss of approximately 5% is observed between 100 °C and 120 °C, indicating the release of water from the hydrate, which then stabilizes at 140 °C. Both spectra indicate that the hydrate’s structure is altered at 100 °C. The peaks at 2.11 THz and 2.43 THz exhibit a gradual shift towards lower frequencies. The observed red shift of the THz absorption peak usually indicates an increase in intermolecular distance, leading to a decrease in vibration frequency [25]. This suggests that the removal of one water molecule results in an increase in intermolecular distances within the unit cell of L-rhamnose monohydrate, thereby causing the crystal structure to become more loosely packed.

**Figure 6:**
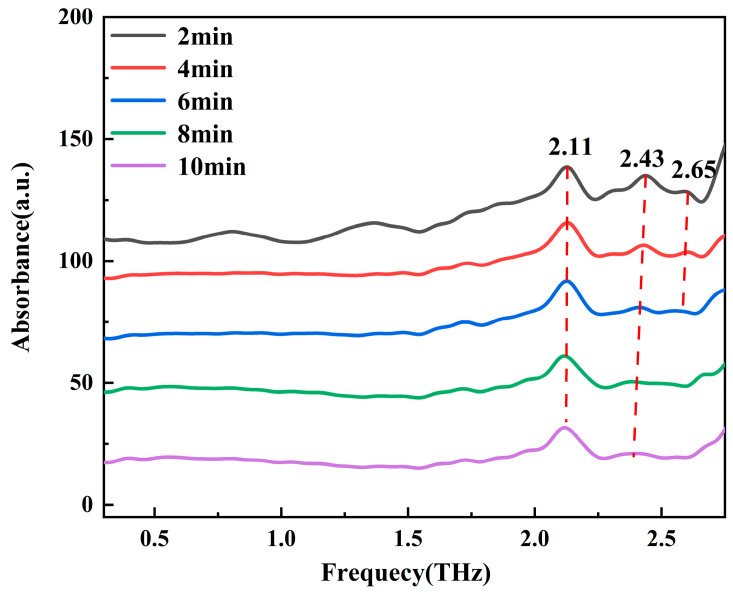
THz spectra of L-rhamnose monohydrate at different times at 100 °C (for clarity, the spectra are vertically shifted).

Figure 6 presents the THz spectra of L-rhamnose monohydrate subjected to dehydration in a vacuum desiccator at 100 °C over various time intervals. The spectra indicate that within 6 min of heating, the absorption peaks at 2.43 and 2.65 THz show a gradual decrease in intensity. After 6 min, these peaks have largely vanished and the spectral curve levels off, suggesting that the dehydration process of L-rhamnose monohydrate is nearly complete by this time. Subsequently, we analyzed the dehydrated L-rhamnose monohydrate and observed that its THz absorption spectrum was identical to that of L-rhamnose, confirming that the dehydration reaction had occurred. During the heating process, L-rhamnose monohydrate loses a water molecule from its unit cell, transforming into anhydrous L-rhamnose.

### 2.5. Molecular Dynamic Calculation

Given the high sensitivity of THz spectroscopy to structural changes, it is essential to complement experimental observations with DFT calculations employing periodic boundary conditions to interpret THz spectra effectively [26]. As water molecules are removed from the crystal structure during dehydration, the molecular arrangement undergoes changes that affect the lattice vibrational modes [27]. These changes are captured precisely by THz spectroscopy. To further investigate the impact of water molecules on the properties of L-rhamnose and its monohydrate before and after dehydration from both molecular structure and intermolecular interaction perspectives, we employed the Perdew–Burke–Ernzerhof (GGA-PBE) exchange-correlation functional in Cambridge Sequential Total Energy Package (CASTEP, version 2022) software for structural optimization and molecular dynamic calculation.

**Figure 7:**
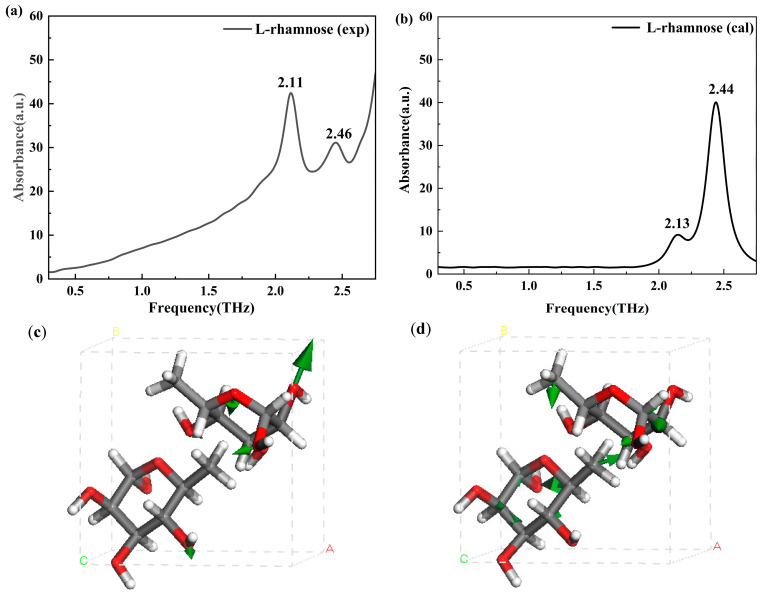
Experimental (**a**) and calculated (**b**) THz spectra, and vibrational modes of L-rhamnose at 2.13 THz (**c**) and 2.44 THz (**d**). ABC represents the unit cell parameters, and the green arrow indicates the vibrational direction at the corresponding THz frequency.

Figure 7a,b show that the experimental THz spectra of L-rhamnose display absorption peaks at 2.11 and 2.46 THz, which align closely with the calculated peaks at 2.13 and 2.44 THz, respectively. As shown in Figure 7c, the peak at 2.11 THz is primarily attributed to the torsional vibrations of the -CH_3_ group and the asymmetric stretching vibrations of the -OH group. Figure 7d shows that the peak at 2.46 THz arises from the in-plane wagging vibrations of the -OH and -O- groups.

**Figure 8:**
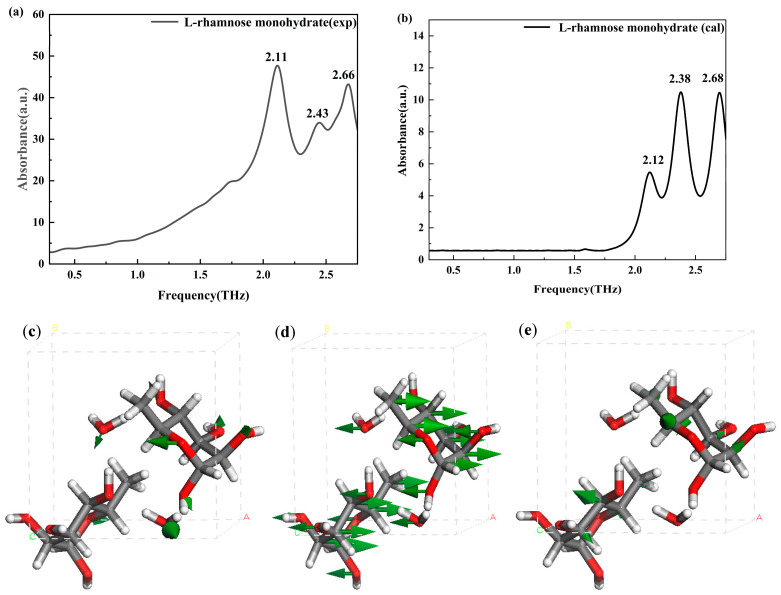
Experimental (**a**) and calculated (**b**) THz spectra, and vibrational modes of L-rhamnose monohydrate at 2.12 THz (**c**), 2.38 THz (**d**), and 2.68 THz (**e**). ABC represents the unit cell parameters, and the green arrow indicates the vibrational direction at the corresponding THz frequency.

Figure 8a,b show that the experimental absorption peak of L-rhamnose monohydrate at 2.11 THz aligns well with the calculated peak at 2.12 THz. As detailed in Figure 8c, this peak is primarily attributed to the combined effects of the wagging vibrations of water molecules, the stretching vibrations of the -OH groups, and the slight wagging of the -O- groups. Similarly, the experimental peak at 2.43 THz corresponds to the calculated peak at 2.38 THz, which is mainly due to the wagging vibrations of the hydrogen atoms on the -OH groups, as shown in Figure 8d. Furthermore, Figure 8e indicates that the absorption peak at 2.68 THz in the calculated spectrum, which corresponds to the experimental peak at 2.66 THz and is attributed to collective molecular vibrations and potentially the translational motion of water molecules.

The detailed vibrational modes for L-rhamnose and its hydrates are summarized in Table 1. The absorption peak observed at 2.66 THz for L-rhamnose monohydrate is primarily attributed to the translational motion of water molecules, whereas no corresponding peak is observed for L-rhamnose within this frequency range. This phenomenon suggests that the incorporation of water molecules into L-rhamnose may alter its lattice structure, resulting in differences in the THz absorption characteristics of L-rhamnose and its monohydrate.

A comparison between the experimental and calculated spectra reveals overall good agreement, though there are noticeable shifts in peak positions. These discrepancies can be attributed to two main factors. First, the theoretical calculations are based on idealized conditions, while real experimental conditions, despite efforts to minimize extraneous influences, hardly replicate these ideal conditions [27]. Second, the crystal structure used in the calculations is optimized theoretically, and THz spectroscopy is highly sensitive to structural variations. Thus, even minor deviations between the actual and theoretical structures can result in subtle variations in spectral features.

## 3. Materials and Experiments

### 3.1. Sample Preparation

L-rhamnose and L-rhamnose monohydrate (purity > 98%) were purchased from Macklin Company and used directly without further purification, both in solid powder form. For Raman and PXRD measurements, the samples were used as received. To investigate the dehydration process of L-rhamnose monohydrate, the sample was placed in a vacuum-drying oven and heated from 20 °C to 140 °C at a controlled temperature gradient of 20 °C. Throughout the heating process, the vacuum pressure and heating rate were strictly controlled. Due to its low absorption in the THz region, polyethylene (PE) was chosen as the substrate for measuring the THz spectra of high-absorption-coefficient samples [28]. We mixed the sample with PE at a mass ratio of 1:5, thoroughly ground this into powder, and compressed it under 60 MPa pressure for 3 min to form a disk with a thickness of 1.1 mm, smooth surface, and no visible cracks.

### 3.2. THz-TDS System

**Figure 9:**
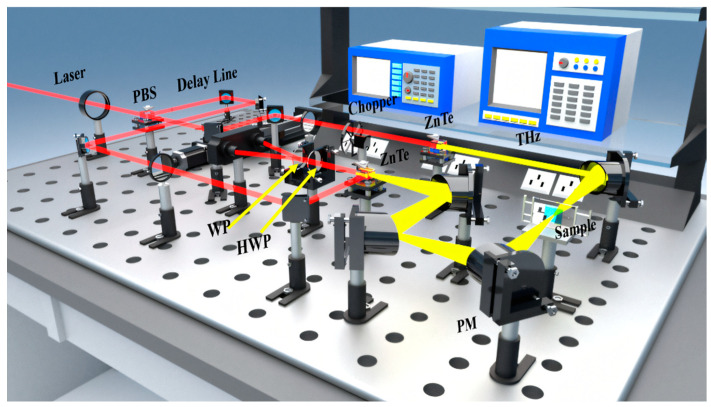
Schematic diagram of THz-TDS system optical path.

The THz-TDS system was employed to measure the THz spectra of L-rhamnose and its monohydrate solids. The system features a self-mode-locking femtosecond laser with a center wavelength of 800 nm, a pulse width of 75 fs, and a high repetition rate of 100 MHz, ensuring high temporal resolution and stability for the measurements. As shown in Figure 9, the laser beam is initially split into pump and detection pulses by a half-wave plate (HWP) and a polarizing beam splitter (PBS). The pump pulse interacts with the ZnTe crystal to generate THz radiation, while the detection pulse traverses the delay system and probes the THz waves through ZnTe crystals. During the measurement process, the prepared sample is positioned at the focal points of two parabolic mirrors. Finally, the obtained optical signal is converted into an electrical signal and collected and processed by a computer. To mitigate the influence of water vapor and other atmospheric interferences, the entire experimental setup was enclosed in a vacuum chamber, ensuring a clean and stable measurement environment.

### 3.3. Raman Spectroscopy Measurement

Raman spectroscopy measurement was carried out using a Renishaw invia Raman spectrometer, which was purchased from Renishaw in the UK. The spectrometer is equipped with a diode pumped solid-state laser as a near-infrared light source with a wavelength of 633 nm. In the experiment, the wavenumber range we used was set between 100–2000 cm^−1^, and the total analysis time for each sample was approximately 5 min. In addition, the working power of the laser is set to about 150 mW, the scanning spatial resolution is 0.1 μm, and the spectral resolution reaches 1 cm^−1^.

### 3.4. PXRD Measurement

PXRD measurement was performed using a D8 Advance diffractometer purchased from Bruker AXS in Germany. The spectrometer is equipped with a Lynxeye array detector, which covers a step scanning range of 3° to 150° and has a peak position accuracy of up to 0.01°. During the experiment, we selected Cu K α radiation as the X-ray source with a wavelength of λ = 1.5406 Å. The detector–sample distance was 90 mm, exposure time was 300 s, and scanning ranged from 10° to 60° at 0.2°/step.

## 4. Methods and Calculations

### 4.1. Absorption Coefficient

Optical parameters play a crucial role in the study of matter and materials. The absorption coefficient is important physical parameter for describing substances. THz-TDS records the time-domain waveform of THz pulses, and through time-domain spectral data, we can obtain other relevant optical information of the sample [29,30].

THz time-domain signal F[E(t)] in free space is:

(1) $$F[E(t)]=E(\omega )=A(\omega )e^{i\varphi (\omega )}$$

where the A(ω) and φ(ω) are the amplitude and phase of THz time-domain signal, respectively.

After fast Fourier transform (FFT), we obtain:

(2) $$\frac{E_{sam}\left(\omega \right)}{E_{ref}\left(\omega \right)}=\frac{A_{sam}\left(\omega \right)}{A_{ref}\left(\omega \right)}e^{i(\varphi_{sam}(\omega )-\varphi_{ref}(\omega ))}=e^{-\alpha (\omega )(d_{sam}-d_{ref})}e^{i\frac{2\pi }{\lambda }(n-1)}$$

where $E_{sam}(\omega )$ and $E_{ref}(\omega )$ are the frequency amplitudes obtained by FFT of the sample signal and the reference signal, $\varphi_{sam}(\omega )$ and $\varphi_{ref}(\omega )$ are the phases of the sample signal and the reference signal, α(ω) is the absorption coefficient, and $d_{sam}$ and $d_{ref}$ are the thicknesses of the sample and the reference signal, respectively. Therefore, according to Formula (2), the absorption coefficient can be obtained as:

(3) $$\alpha \left(\omega \right)=-\frac{d}{2}\ln\left\{\frac{{\left[n\left(\omega \right)+1\right]}^{2}}{4n\left(\omega \right)}\left|\frac{E_{sam}\left(\omega \right)}{E_{ref}\left(\omega \right)}\right|\right\}$$

### 4.2. Simulation Calculation

Quantum mechanics provides the theoretical foundation for explaining intermolecular forces, electronic structures, material spectra, and electronic spectra. This theoretical framework has been extensively applied in THz spectroscopy research, offering fundamental insights into material structures and chemical reactions [6]. In this study, the lattice structure was sourced from the CCDC. The corresponding lattice parameters for L-rhamnose are a = 7.801 Å, b = 8.022 Å, c = 6.880 Å, α = 90°, β = 90°, γ = 90°, while for its monohydrate, the parameters are a = 7.901 Å, b = 7.921 Å, c = 6.670 Å, α = 90°, β = 95.52°, γ = 90° (CCDC 1249284) [31]. Geometric optimization and energy calculations of the crystals were performed using the CASTEP module in Materials Studio (MS) software (Accelrys Ltd., version 2022, San Diego, CA, USA) [32]. In this framework, the GGA-PBE exchange-correlation functional [33,34] was employed, along with the Tkatchenko–Scheffler (TS) dispersion correction method [35,36]. Ultrafine calculation settings were used, and the traditional conservative pseudopotential was selected [37]. Notably, the plane-wave cutoff energy was set to 830 eV with the K-point sampling in the Brillouin zone set to Fine 3 × 3 × 4, and the basis set used was DNP+. All vibrational calculations were performed on geometrically optimized structures to ensure the accuracy of the energy calculations.

## 5. Conclusions

In conclusion, this study utilized THz-TDS technology and DFT calculations to investigate the low-frequency vibrations of L-rhamnose and its monohydrate. The findings indicate that THz spectroscopy provides superior qualitative identification of L-rhamnose and its monohydrate compared to Raman and PXRD techniques within the 0.3–2.75 THz frequency range. By utilizing THz spectroscopy to study the dehydration temperature of L-rhamnose monohydrate, we found that under vacuum conditions, the monohydrate can be fully dehydrated within 6 min at 100 °C. Additionally, solid-state DFT calculations revealed that the presence of a single molecule of crystalline water in L-rhamnose monohydrate significantly impacts the THz absorption spectrum through its influence on molecular vibrations. These results not only confirm the effectiveness of THz-TDS technology in the analysis of carbohydrates and their hydrates but also provide valuable insights for monitoring the dehydration process of hydrates. This work highlights the capability of THz spectroscopy to offer detailed structural information and enhances our understanding of the vibrational characteristics associated with hydration changes.

## Figures and Tables

**Table 1:** Spectral characteristics and corresponding vibration modes of L-rhamnose and its hydrates.

Sample	Exp. (THz)	Cal. (THz)	Vibration Mode Description
L-rhamnose	2.11	2.13	twisting of -CH_3_ and asymmetric stretching of -OH
	2.46	2.44	in plane oscillation of -OH and -O-
L-rhamnose monohydrate	2.11	2.12	oscillation of water molecules, stretching of -OH on the interstitial C atom, and slight oscillation of -O-
	2.43	2.38	swing of H atom on -OH
	2.66	2.68	collective vibration of molecules and the translation of water molecules

## Data Availability

The data used to support the findings of this study are available from the corresponding author upon request.
